# Light Energy Conversion Surface with Gold Dendritic Nanoforests/Si Chip for Plasmonic Polymerase Chain Reaction

**DOI:** 10.3390/s20051293

**Published:** 2020-02-27

**Authors:** Hung Ji Huang, Yu-Cheng Chiang, Chia-Hsien Hsu, Jyh-Jian Chen, Ming-Hua Shiao, Chih-Chieh Yeh, Shu-Ling Huang, Yung-Sheng Lin

**Affiliations:** 1 Taiwan Instrument Research Institute, National Applied Research Laboratories, Hsinchu 30076, Taiwan; hjhuang@narlabs.org.tw (H.J.H.); mhshiao@narlabs.org.tw (M.-H.S.); 2 Department of Food Nutrition and Health Biotechnology, Asia University, Taichung 41354, Taiwan; honda224@asia.edu.tw; 3 Institute of Biomedical Engineering and Nanomedicine, National Health Research Institutes, Miaoli 35053, Taiwan; chsu@nhri.org.tw; 4 Department of Biomechatronics Engineering, National Pingtung University of Science and Technology, Pingtung 91201, Taiwan; chaucer@mail.npust.edu.tw; 5 Department of Chemical Engineering, National United University, Miaoli 36063, Taiwan; a0968022350@gmail.com (C.-C.Y.); simone@nuu.edu.tw (S.-L.H.)

**Keywords:** polymerase chain reaction, *Salmonella* spp. DNA, gold, dendritic, silicon, plasmon

## Abstract

Surfaces with gold dendritic nanoforests (Au DNFs) on Si chips demonstrate broadband-light absorption. This study is the first to utilize localized surface plasmons of Au DNFs/Si chips for polymerase chain reaction (PCR) applications. A convenient halogen lamp was used as the heating source to illuminate the Au DNFs/Si chip for PCR. A detection target of *Salmonella* spp. DNA fragments was reproduced in this plasmonic PCR chip system. By semi-quantitation in gel electrophoresis analysis, the plasmonic PCR with 30 cycles and a largely reduced processing time provided results comparable with those of a commercial PCR thermal cycler with 40 cycles in more than 1 h. In the presence of an Au DNFs/Si chip, the plasmonic PCR provides superior results in a short processing time.

## 1. Introduction

Light conversion/redistribution surfaces are essential for harvesting thermal [1,2,3,4,5,6,7,8,9,10], optical [11], electrical [12,13,14], photocatalytical or photochemical [15,16], or other types of energy from electromagnetic waves. Using artificially created surface structures on Si, the absorption efficiency and, in turn, the processing efficiency of solar cells can be enhanced to a large extent [12]. The plasmonic effect can increase the light absorption of a metal that originally reflects light. The “optical black hole” can be achieved using metamaterial [1]. The plasmonic light–energy conversion efficiency also strongly affects the processing efficiency of applications, such as solar-light-enhanced methanol oxidation reactions [14,15], water heating [2], and photocatalytic reactions [16]. The simplified energy transformation of harvested light in metals is known as electromagnetic heating [2]. The sizes and shapes of nanoparticles (NPs) [4,17] or networks [5] affect the efficiency of such heating. The localized high temperature generated by plasmonic heating can be used for photothermal nanoblades [3] and targeted therapy for dysfunctional cells [6,7]. This paper discusses the use of surfaces of dendritic nanoforests (DNFs) on a Si substrate [13,14] to enhance plasmonic light-to-heat conversion for rapid polymerase chain reactions (PCRs).

PCR was developed by Kary Mullis [18] in 1983 and is widely used in molecular biology to generate exponentially amplified copies of a specific DNA segment for a broad variety of applications, including biomedical research and criminal forensics [8,19,20,21,22]. Most PCR methods rely on thermal heating and cooling cycles to permit DNA melting and enzyme-driven DNA replication. PCR rapidly delivers results with high sensitivity of a specific product for sequencing, cloning, and analysis. Microfluidic lab-on-a-chip or micro total analysis systems (μ-TAS) [23] are potential technologies to miniaturize the conventionally used equipment in PCR. Northrup et al. developed the first PCR chip, which led to the development of many advanced microfluidic PCR chips [21,22]. Microfluidic PCR chips can be classified into two types [24,25]: chamber stationary PCR chips [26,27,28,29] and flow-through PCR chips [30,31,32]. Ongoing extensive developments of PCR microfluidic chips are focused on technologies that facilitate shorter processing times, are cheaper, and allow processing of multiple samples simultaneously.

In PCR, the heating and cooling processes are necessary in the repeated two or three-temperature cycles to gradually amplify copies of a specific DNA segment. The equipment is a typical combination of thermal resist, thermal electric (TE) heater/cooler, heat sink, and fans. Heating with illuminating light is a clean and fast method for PCR. Chen and Hsieh used an infrared (IR) lamp for DNA amplification using an oscillatory thermocycler for PCR of 385-bp *Coxiella burnetii* DNA in a poly (methyl methacrylate) (PMMA) tank [27]. Highly efficient plasmonic light harvest of visible light is recommended for less damage to DNA and a rapid increase in temperature [8,10,33,34].

Some studies have utilized the benefits of surface plasmons to speed up PCR applications. Plasmonic heating can largely and rapidly increase the temperature of metal NPs and is especially favorable as a heat source in the so-called plasmonic PCR. Son et al. used a blue LED (447.5 nm, 890 mW) to heat the 10-μL DNA test solution (0.1 ng/μL) in a hole covered with an Au thin film and processed 30 PCR cycles in 5 min [8]. Li et al. used near IR laser (808 nm, 460 mW) to heat the DNA test solution containing suspended Fe_3_O_4_ NPs to 90 °C in 15 s. The test solution cooled to 65 °C in approximately 15 s after the IR laser was turned off, and 30 PCR cycles were processed in 7 min [33]. Under illuminating light, Au nanomaterials can largely increase the surface plasmons response. Kim et al. used IR laser (808 nm, 5.6 W/cm^2^) to heat the test solution containing suspended Au nanorods and processed 30 PCR cycles in 30 min [34]. Lee et al. used IR laser (846 nm, 8.5 W) to heat the test solution containing suspended Au bipyramid nanorods and processed 40 PCR cycles in 40 min [10]. Roche et al. used IR laser (808 nm, 2 W/cm^2^) to heat a PCR solution containing suspended Au nanorods and processed 30 PCR cycles in 1 min [9].

Plasmonic light-to-heat energy conversion with Au NPs suspended in a solution results in ultra-high-speed plasmonic PCR with a short processing time. However, metal NPs do not easily separate from the sample solution during recovery and may result in aggregation and erroneous quantitative analysis of DNA fragments. The holes in PMMA covered with an Au thin film may overcome the problem of high reflectivity even in a typical volume plasmon or plasma wavelength region. Only red or NIR light may be used for the Au nanorods, whereas only blue light or lights with shorter wavelengths may be used for the Au thin film. Part of light energy is lost during PCR. In this study, Au DNFs/Si with broadband visible light absorption are presented as an alternative heat source for potential high-speed, clean, and accurate PCR applications.

## 2. Materials and Methods

**Figure 1:**
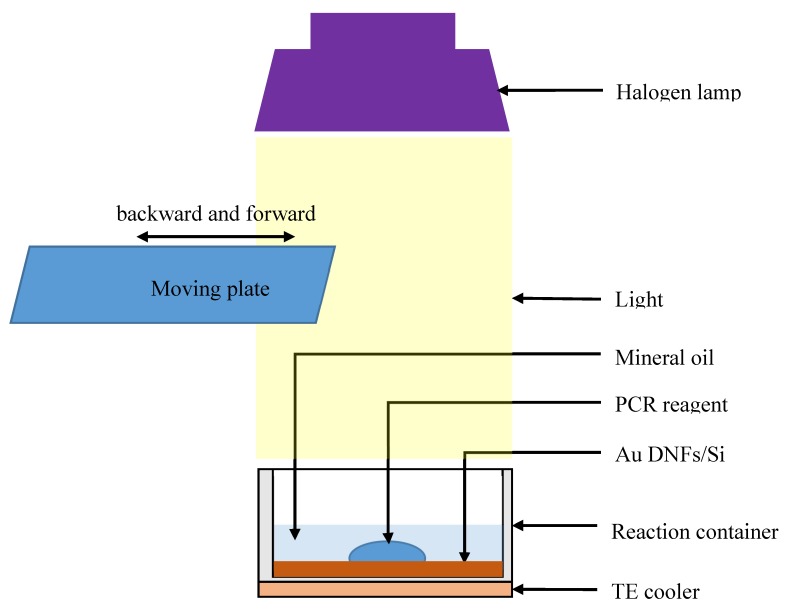
Schematic illustration of the PCR system containing the Au DNFs/Si chip, reaction container, TE cooler, moving plate, and halogen lamp.

In this study, the plasmonic PCR processed with Au DNFs/Si was processed using a self-made intermittent light-illumination facility (Figure 1). The Au DNFs/Si chip was placed in a 12 mm × 12 mm × 10 mm (length × width × height) reaction container of 1 mm thickness made by using the 3D printing method (Laser melting, Renishaw AM250). The reaction container held a thermal couple to depict the temperature of the mineral oil covering the PCR reagent for monitoring and feedback control. In addition to 25 μL of PCR reagent, 200 μL of mineral oil was added to the container to cover the PCR reagent to avoid water evaporation. A convenient 250-W halogen lamp with a 12° light-illumination cone served as a homogeneous photon energy source within a 6 cm circular area. An aluminum moving plate moved back and forth by a step motor to intermittently provide photon energy. The photon energy absorbed by the Au DNFs/Si chip, solution, and the metal container was heated under light illumination. The light illumination turned on and off in response to the aluminum moving plate when the monitoring temperature reached the low and high limits of set temperatures. The TE cooler was turned on to fast cool the reaction container during the cooling process. 

A halogen lamp with an aluminum reflector was used as a light source to deliver a broadband visible light for heating processes in the PCR cycles. The light absorption by Au DNFs on the Si substrate can enhance the utilization of a very broadband visible light [15,16,35]. This implies that a broadband light source from a typical halogen lamp, a narrow band solid-state light source, LED, or laser are all suitable for heating the Au DNFs/Si. It is not necessary to find the specific method to generate NPs with a specific size to meet the demand of the used light source.

The synthesis of Au DNFs on the Si substrate followed a fluoride-assisted galvanic replacement reaction (FAGRR) [13,14,35]. The Si substrate was cleaned by sequentially using solutions of acetone, methyl alcohol, and deionized (DI) water in an ultrasonic bath for 5 min each and then dried using a nitrogen shower and baking at 120 °C. The nature oxide thin film on the Si substrate was then removed using a buffered oxide etch (BOE) etchant. The FAGRR steps are generally as follows: anode: Si + 6F^−^ → SiF_6_^2−^ + 4e^−^; cathode: AuCl_4_^−^ + 3 e^−^ → Au + 4 Cl^−^

In total, 8 mL of a 29.56 mM HAuCl_4_ solution was mixed with 8 mL of a BOE solution to synthesize DNFs on the Si substrate in 3 min. The DNFs/Si chip was then cleaned using DI water and dried using a nitrogen shower and baking at 120 °C. Characterization analysis of the obtained sample was carried out using scanning electron microscope (SEM) coupled with energy-dispersive X-ray spectroscopy (EDS), Hitachi SU8000, Japan.

In the PCR experiments, a 2-μL DNA fragment (*Salmonella* spp.) was mixed with 2 μL of 2.5 mM dNTPs, 2.5 μL of 10 × buffer, 1 μL of 10 μM F primer, 1 μL of 10 μM R primer, 0.2 μL of 2 U Prozyme, and 16.3 μL of DI water to obtain 25 μL of the PCR test solution. Primers S15S (5-AACGGACGGTGATCGTTAAA-3) and S15A (5-AGGGCGTTAAGCTTGCGC-3) were added to the test solution. The stable cyclic-temperature PCR protocol for genomic DNA amplification was used in this study. After the PCR experiments, 3 μL of test solution was retrieved from the container and processed using electrophoresis in 2% agarose 0.5 × Tris-acetate-EDTA (TAE) buffer. The results were imaged using a UV box after SafeView DNA staining.

## 3. Results and Discussion

**Figure 2:**
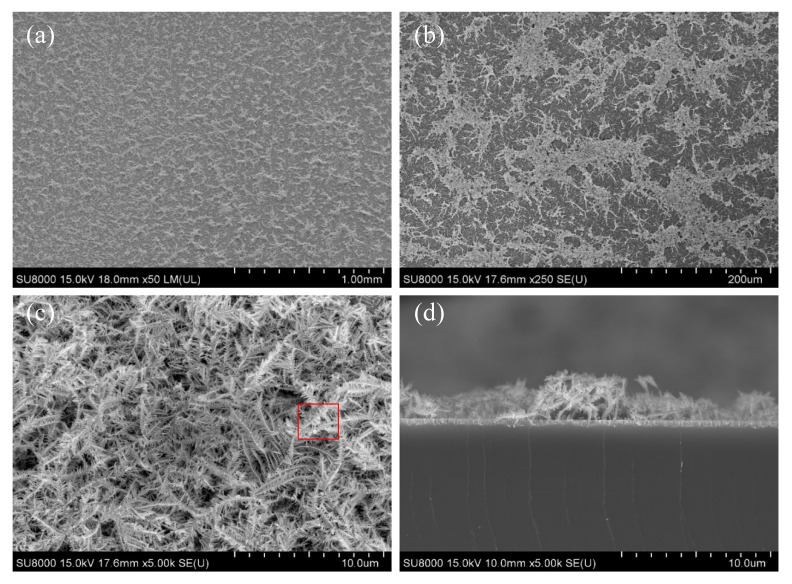

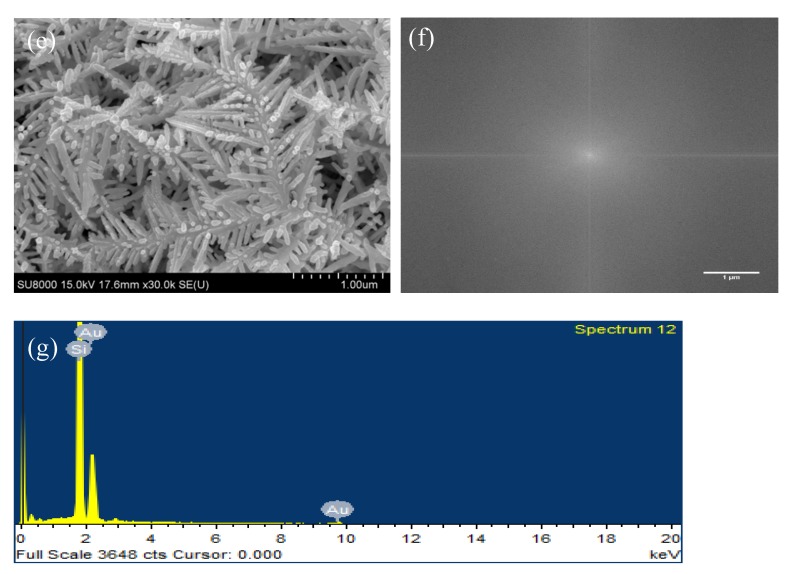
SEM pictures of the Au DNFs/Si chip. (**a**) 50 × top view; (**b**) 250 × top view; (**c**) 5000 × top view; (**d**) 5000 × side view; (**e**) zoom-in image for FFT; (**f**) FFT image of (**e**); (**g**) EDS spectrum of the marked region in (**c**).

After 3 min of growth time in FAGRR, the Au DNFs homogeneously grew on the Si substrate (Figure 2a,c) and the SEM picture presents that the height of the DNFs increased to approximately 3.5 μm (Figure 2d). The plasmonic response at the nanoscale is largely dependent on the material, size, shape, and structure of nanomaterials [4,5]. A homogeneous size distribution of nanostructures or nanomaterials can provide a broadband plasmonic response and light-to-heat conversion property. Therefore, the synthesized Au DNFs were further studied by applying fast Fourier transformation (FFT) [36] on the SEM image/data by using ImageJ. The zoomed-in image (Figure 2e) showing the nanometer-sized details of nanostructures was acquired as the source image for FFT. The FFT image (Figure 2f) shows a circular Gaussian-like broad homogeneous distribution of spatial frequency locates at the center. No specific highs or lows in the FFT image of the SEM data imply homogeneity in the distribution of sizes and gaps of the synthesized Au DNFs. This explains why the Au DNFs/Si present a broad homogeneous light absorption, suitable to process the light-to-heat conversion for heating the test solution in PCR. EDS (Figure 2g) shows comparable signals of Au and Si because the Au DNFs are thick. By analysis through inductively coupled plasma spectrometry, 0.54 mg of Au DNFs was synthesized on the Si substrate on an area of 1 cm × 1 cm. These results agreed with the results of our previous work on Au DNF synthesis [15,16,35].

**Figure 3:**
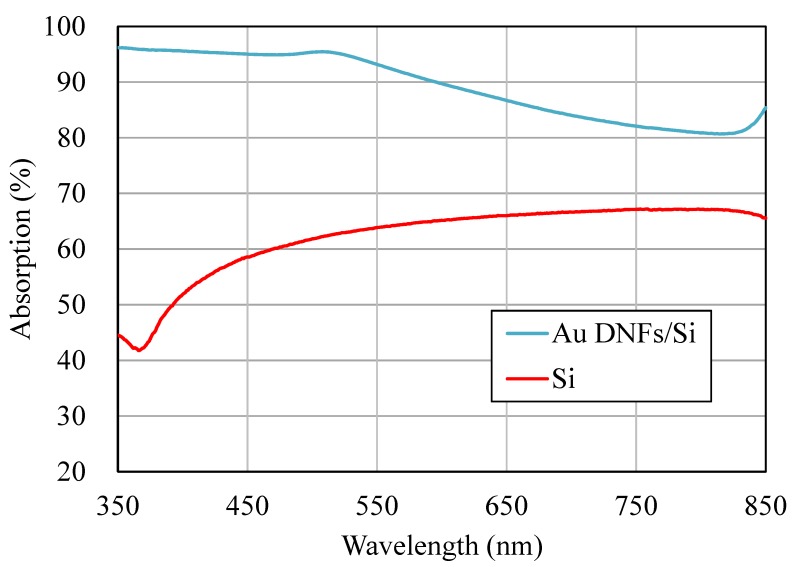
Absorption spectra of Au DNFs/Si and Si chips.

The key to using maximum light energy is having a broad absorption band of incident light. The absorption of Au DNFs on the Si substrate can enhance the utilization of very broadband visible light from less than 350 nm to greater than 850 nm (Figure 3). The size of the dense gaps between leaves of the nano trees in DNFs is approximately a submicron structure and has a wavelength similar to that of visible-to-near infrared (VIS-to-NIR) light. This implies that the thick DNFs on the Si substrate can absorb a large amount of broadband VIS-to-NIR light (Figure 3) and that the illuminating light from the halogen lamp can be converted to heat through plasmonic heating processes. The reflected light absorption ratio of the VIS-to-NIR light by the Si substrate is less than 65%. A metal layer typically increases the reflection of illuminating light. However, the Au DNFs/Si largely increase the absorption of the reflected light to at least 80%. Absorption of the reflected light increases to 95% when the wavelength of the reflected light is less than 500 nm, which is the plasma wavelength of Au. Some studies have used the roughening process to largely decrease the reflection of illuminating light and increase the processing efficiency in applications of solar cells [12]. However, it is not suitable for applications in plasmonic PCR as the chemical activity of Si is high and unexpected reactions may occur with the reactants, leading to an error signal in PCR.

**Figure 4:**
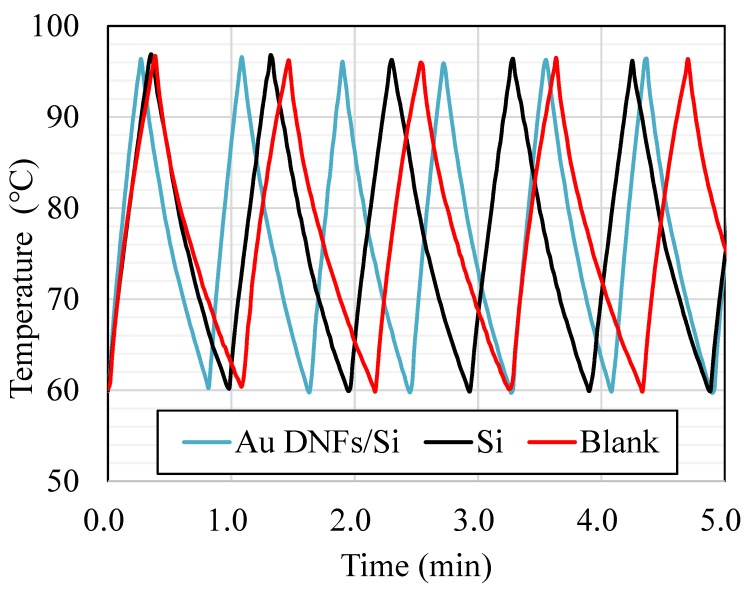
Cyclic temperature recorded in the PCR system.

Additionally, an automatic temperature cycling testing process of mineral oil in the container was processed to examine the function of the self-made intermittent light-illumination facility (Figure 4). The system automatically detected the container temperature and turned the TE cooler on and off, and the moving plate responded to maintain the container temperature between 60 and 97 °C. The thermal cycling of heating and cooling in the PCR process was feasible because of the stable and regular temperature cycle. The average cycling time for the system with the Au DNFs/Si chip was approximately 45 s. The Si chip and blank control without any chip took more time to have the same cyclic temperature.

**Figure 5:**
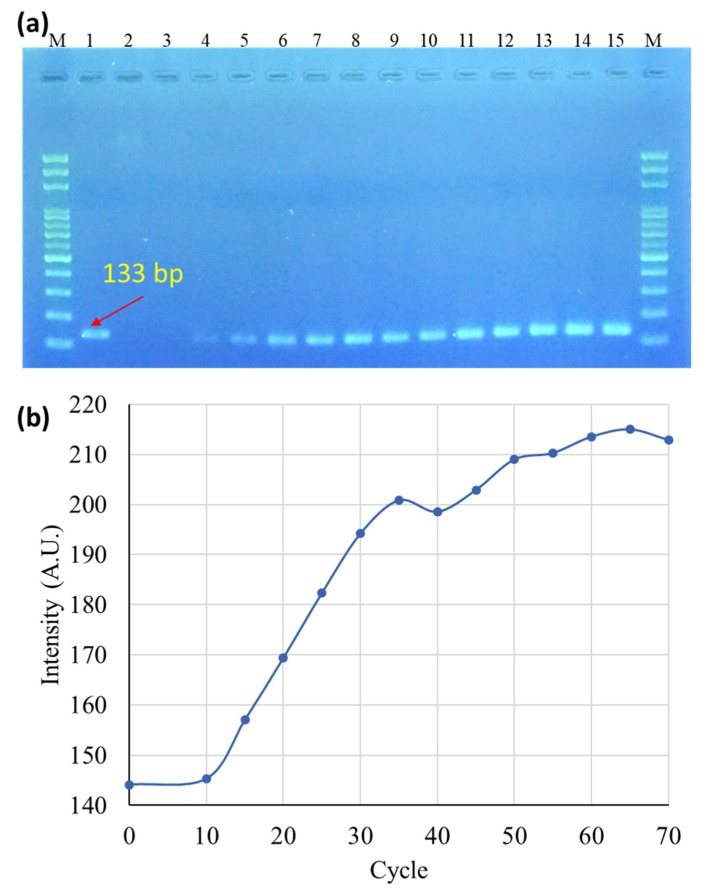
(**a**) Gel electrophoresis of PCR products. Lane M: 100 bp ladder marker, Lane 1: PCR product obtained using the commercial PCR thermal cycler, Lane 2: *Salmonella* spp. template DNA before PCR, Lanes 3–15: PCR products from the Au DNFs/Si chip after 10, 15, 20, 25, 30, 35, 40, 45, 50, 55, 60, 65, and 70 PCR cycles. (**b**) Intensity analysis of target bands in gel electrophoresis results.

The products obtained through PCR were compared using gel electrophoresis (Figure 5). Lane M is the 100-bp ladder marker for reference. Lane 1 is the PCR product of *Salmonella* spp. template DNA after 40 cycles using a commercial PCR thermal cycler, the Applied Biosystems 2720 Thermal Cycler (Perkin-Elmer Corporation, Norwalk, CT, USA). Lane 2 is *Salmonella* spp. template DNA before PCR. Lanes 3–15 are PCR products obtained from the Au DNFs/Si chip after 10, 15, 20, 25, 30, 35, 40, 45, 50, 55, 60, 65, and 70 PCR cycles, respectively. By analysis of the semi-quantitation method, fluorescence intensity measured using ImageJ in Lane 1 was between those in Lanes 5 (25 cycles, 18.75 min) and 6 (30 cycles, 22.5 min). This indicated that the plasmonic PCR with 30 cycles and a 22.5-min processing time can provide results comparable with those obtained using the commercial PCR thermal cycler with 40 cycles and more than 1 h processing time. Using the proposed plasmonic PCR method, the processing time was largely reduced, and processing accuracy was maintained.

**Figure 6:**
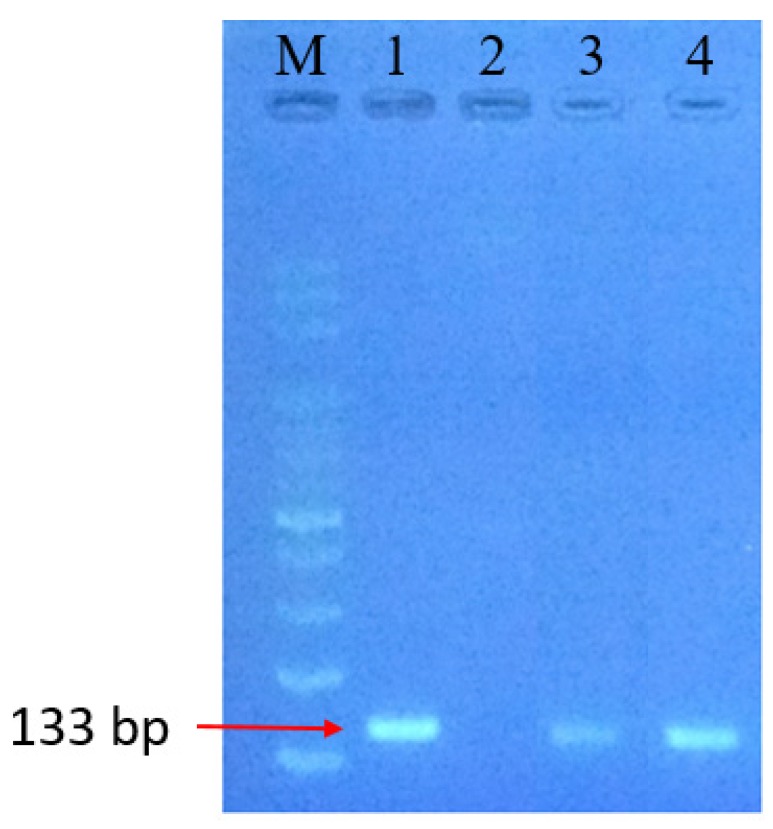
Gel electrophoresis of PCR products processed with and without the Au DNFs/Si chip. Lane M: 100-bp ladder marker, Lane 1: PCR product obtained using the commercial PCR thermal cycler, Lane 2: *Salmonella* spp. template DNA before PCR, Lanes 3: PCR product without the Au DNFs/Si chip, Lanes 4: PCR product with the Au DNFs/Si chip.

An additional PCR test by a commercial machine and this self-made PCR reactor system present the superior function of the Au DNFs/Si chip in the plasmonic PCR. The obtained PCR products were compared using gel electrophoresis (Figure 6). Lane M is the standard reference, and Lane 1 is the PCR product of *Salmonella* spp. template DNA after 40 cycles using a commercial PCR thermal cycler. Lane 2 is *Salmonella* spp. template DNA before PCR and has no signal intensity. Lanes 3 and 4 are PCR products from 30 PCR cycles without and with the Au DNFs/Si chip, respectively. The results indicate that the 30 cycles of plasmonic PCR by the Au DNFs/Si chip system had a compatible fluorescence intensity (Lane 4) with that of 40 cycles PCR by the commercial machine (Lane 1), agreeing with the result shown in Figure 5. However, the 30 cycles PCR without the Au DNFs/Si chip show apparent lower fluorescence intensity (Lane 3) than that processed with Au DNFs/Si chip (Lane 4). The thermal cycle processed by only light illumination on the PCR reaction container can also obtain PCR products but has an even lower efficiency. Therefore, the plasmonic PCR with an Au DNFs/Si chip provides superior efficiency in PCR results compared with the conventional thermal machine and the light illumination method. The broadband light absorption is essential for the plasmonic PCR with the Au DNFs/Si chip.

The light-to-heat energy conversion can be processed using various types of plasmons, such as volume plasmons, surface plasmons, and localized surface plasmons. Volume plasmons are related to the nature frequency or plasma frequency of the highest response of bulk metal to external electromagnetic waves or light. Surface plasmons are the induced waves at the metal–dielectric interface. Localized surface plasmons are generated on nanostructures, such as NPs, nanorods, edges, or air gaps, on a thin film or bulk metal. Plasmons are a collective motion of free electrons induced by an external electromagnetic wave. The plasmon–phonon interaction, or simply, resistance of the motion of free electrons, results in a tiny area with an ultrahigh temperature and a high electromagnetic response. This high temperature or high electromagnetic field response area is called a hot spot and presents ultrahigh light-to-heat energy conversion points. The size and shape of metal nanostructures affect the light-to-heat energy conversion in one- to three-dimensional plasmonic confinements. Plasmonic heating of DNFs results in more couplings of light that mainly arise from the complex size distribution of the surface area, gaps, and networks where a large electromagnetic field and numerous hot spots are induced. Plasmonic heating can largely and rapidly increase the temperature of metal NPs and is especially favorable as a heat source in relative applications, e.g., the so-called plasmonic PCR in this study.

## 4. Conclusions

A surface with DNFs on the Si substrate was used to demonstrate the enhancement of plasmonic broadband light-to-heat conversion for PCR with a short processing time. No specific highs or lows in the FFT image of the SEM data implied homogeneity in the distribution of sizes and gaps of the synthesized Au DNFs that led to a broader and smoother light absorption band. The synthesized Au DNFs on the Si substrate thus presented a fast light-to-heat conversion method for increasing the temperature of the test solution. The plasmonic PCR can provide results comparable with those of a commercial PCR thermal cycler, with largely decreased cycle numbers and processing time. In the presence of Au DNFs/Si, the plasmonic PCR can provide superior results in a short processing time and can thus have advanced applications in the future.
